# Differential Distribution and Determinants of Ammonia Oxidizing Archaea Sublineages in the Oxygen Minimum Zone off Costa Rica

**DOI:** 10.3390/microorganisms7100453

**Published:** 2019-10-15

**Authors:** Yanhong Lu, Xiaomin Xia, Shunyan Cheung, Hongmei Jing, Hongbin Liu

**Affiliations:** 1 SZU-HKUST Joint PhD Program in Marine Environmental Science, Shenzhen University, Shenzhen 518061, China; ylubf@connect.ust.hk; 2 Department of Ocean Science, The Hong Kong University of Science and Technology, Hong Kong, China; sycheungab@connect.ust.hk; 3 Key Laboratory of Tropical Marine Bio-resources and Ecology, South China Sea Institute of Oceanology, Chinese Academy of Sciences, Guangzhou 510220, China; xiaxiaomin@scsio.ac.cn; 4 CAS Key Laboratory for Experimental Study under Deep-sea Extreme Conditions, Institute of Deep-sea Science and Engineering, Chinese Academy of Sciences, Sanya 572000, China; hmjing@idsse.ac.cn; 5 Hong Kong Branch of Southern Marine Science & Engineering Guangdong Laboratory, The Hong Kong University of Science and Technology, Hong Kong, China

**Keywords:** ammonia oxidizing archaea, *amoA* gene, ecotype, deep oxygen minimum zone, Costa Rica Dome

## Abstract

Ammonia oxidizing archaea (AOA) are microbes that are widely distributed in the ocean that convert ammonia to nitrite for energy acquisition in the presence of oxygen. Recent study has unraveled highly diverse sublineages within the previously defined AOA ecotypes (i.e., water column A (WCA) and water column B (WCB)), although the eco-physiology and environmental determinants of WCB subclades remain largely unclear. In this study, we examined the AOA communities along the water columns (40–3000 m depth) in the Costa Rica Dome (CRD) upwelling region in the eastern tropical North Pacific Ocean. Highly diverse AOA communities that were significantly different from those in oxygenated water layers were observed in the core layer of the oxygen minimum zone (OMZ), where the dissolved oxygen (DO) concentration was < 2μM. Moreover, a number of AOA phylotypes were found to be enriched in the OMZ core. Most of them were negatively correlated with DO and were also detected in other OMZs in the Arabian Sea and Gulf of California, which suggests low oxygen adaptation. This study provided the first insight into the differential niche partitioning and environmental determinants of various subclades within the ecotype WCB. Our results indicated that the ecotype WCB did indeed consist of various sublineages with different eco-physiologies, which should be further explored.

## 1. Introduction

Nitrification is a microbe-mediated sequential oxidation of ammonia to nitrate that interconnects the biological nitrogen fixation and nitrogen loss processes in the nitrogen cycle. There is increasing recognition of the importance of nitrification, both in nitrogen regeneration which fuels the primary production [1,2] and in the production of a highly potent greenhouse gas (N_2_O) [3]. Ammonia oxidation, the first and rate-limiting step of nitrification, was once considered to be only performed by ammonia oxidizing bacteria (AOB) [4]. However, later studies discovered that ammonia oxidizing Thaumachaeota (AOA) were the major ammonia oxidizers in the ocean, and they are distributed ubiquitously in marine water columns, estuaries, sediments [5,6,7] as well as marine oxygen minimum zones (OMZs) [5,8,9,10]. By quantifying ammonia mono-oxygenase (*amoA*) gene fragments with quantitative polymerase chain reaction (qPCR), previous studies conducted in various marine environments (including the California current, North Sea, Monetary Bay and the North Pacific) showed that AOA were several orders of magnitude more abundant than AOB in oceanic waters [5,11,12]. Culture-based studies have reported that AOA have a higher affinity to ammonia than AOB [13] and that they use a highly energy-efficient carbon fixation pathway [14], which suggests the dominance of AOA in the nitrogen cycle of oligotrophic oceanic waters. 

Globally, AOA communities have been found to mainly consist of 3 groups, water column A (WCA), water column B (WCB) and SCM1-like; with WCA and WCB being particularly dominant in oceanic water columns [5,6,15]. The WCA and WCB groups are defined as shallow-water and deep-water ecotypes, respectively [6], based on their differential vertical distributional pattern along the water column. It has been proposed that the distribution pattern of these two ecotypes is governed by physicochemical conditions, such as ammonium, light, and oxygen concentration [15,16,17,18,19,20]. Recently, a number of subclades of WCA and WCB were defined in a global database, and the vertical and horizontal distribution patterns of the subclades of WCA have been related to different environmental conditions (e.g., within the WCA I subclade, WCAI-A prefers shallow water with a low concentration of organic nutrient and WCAI-C are mainly distributed in mesopelagic zones with low temperatures) [15]. However, due to the homogeneous physicochemical conditions in the mesopelagic zone of the stations sampled in the previous study, the dynamics and environmental determinants of WCB subclades are still unclear [15]. A recent global synthesis of AOA phylogeny pointed out that AOA clades, which contributed over 55% *amoA* phylotypes to the global database, lack cultivated representatives [21]. This has greatly hindered our understanding of the roles and life strategies of the abundant AOA in the global biogeochemical cycle. This situation is the most serious issue in the marine ecosystem. To date, only one subclade (WCAI) of WCA and the SCM1-like group have cultivated representatives from the ocean (*Ca*. *Nitrosopelagicus brevis* CN25 and *Nitrosopumilus maritimus* SCM1, respectively) [21,22,23], and our understanding of the WCB is still heavily reliant on its eco-physiological characteristics related to its distribution and the environmental conditions of the habitats. 

Traditionally, aerobic ammonia oxidation was not considered to be directly linked with the anaerobic nitrogen loss process (e.g., denitrification and anammox), because both processes were supposed to take place under disparate oxygen levels, that is, ammonia oxidation requires oxygen whereas denitrification and anammox are sensitive to oxygen concentration [8,10]. Depletion of oxygen and ammonia is believed to limit AOA in the OMZ. However, there has been increasing evidence of the activity and importance of ammonia oxidation in OMZs. Several studies have reported archaeal *amoA* gene in DNA and at the RNA level, providing molecular evidence of ammonia oxidation in the OMZs [5,24,25,26]. A recent study detected active ammonia oxidation in the core of a seasonal OMZ off Concepcion, Chile, which implied that ammonia oxidation competes with anammox for substrate, and hence, acted as a control factor for nitrogen loss [27]. Although ammonia oxidation can occur in several nanomole oxygen, its activity was found to be highly dependent on oxygen level [27]. Considering the high diversity of marine AOA and the strong relationship between ammonia oxidation activity and oxygen level leads to speculation that the unique environment in the OMZ may select low-oxygen tolerating AOA groups and result in a unique AOA community. However, the previous relevant studies used low resolution biomarkers (16s *rRNA* gene), short fragments of *amoA* gene (70 bp) or clone library analysis of *amoA* genes (limited sequence number per sample) to analyze the phylogeny of AOA, which were inadequate for reconstructing the AOA communities in the OMZs and to provide sufficient phylogenetic information [9,10,27]. 

The Costa Rica Dome (CRD) is a wind-driven upwelling system that contains the largest OMZ in the eastern tropical North Pacific (ETNP) Ocean [28]. High productivity in the euphotic layer continuously supplies sinking organic matter to subsurface water, resulting in a permanent OMZ at depth of 400–700 m that is much deeper than other major OMZs in the eastern tropical South Pacific (ETSP) and Arabian Sea [28]. In this study, we have for the first time used high-throughput 454 pyrosequencing to unravel the highly diverse AOA community in the OMZs off the CRD. Instead of simply distinguishing the AOA phylotypes as ecotypes WCA and WCB, we analyzed and compared the fine-scale diversity of AOA at the euphotic zone, upper mesopelagic water above OMZ, OMZ core, lower mesopelagic zone below OMZ and bathypelagic water, following the refined definition of AOA subclades in recent studies [15,29]. We hypothesized that (1) the unique physicochemical conditions in the OMZ selected unique low-oxygen tolerant phylotypes of AOA and results in distinct AOA communities between OMZ and non-OMZ waters off the CRD, and (2) the OMZ selected AOA phylotypes founded in the CRD are shared by geographic distant OMZs in global oceans. Taking advantage of the steep environmental gradient in the mesopelagic zone off the CRD, we also aimed to understand the eco-physiology of the poorly understood WCB subclades.

## 2. Materials and methods

### 2.1. Sample Collection and Environmental Factor Measurement

**Figure 1:**
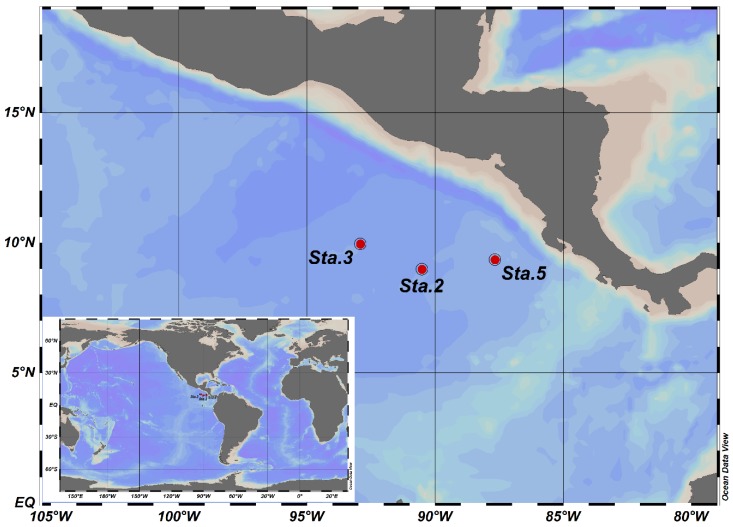
Sampling location in the Costa Rica Dome.

The study was conducted in the CRD during the FLUZiE cruise on board R/V Melville from June to July 2010 [28]. The samples for this study were collected from stations located in Cycle 2, 3, and 5 of the CRD-FLUZiE cruise, in which cycle 2 (station 2) was located within the upwelling dome, cycle 3 (station 3) was located outside the dome and cycle 5 (station 5) was located at the edge of the dome (Figure 1). Water samples were collected from Niskin bottles together with the basic hydrographic data (salinity, temperature, depth and dissolved oxygen) from a conductivity-temperature-depth (CTD, Sea-Bird Electronics) instrument. Ammonium concentrations were measured immediately on board using the orthophthaldehyde (OPA) method [30]. Nitrite concentrations were also determined on board using the Greiss-Ilosvay colorimetric method [31]. For molecular samples, 1 L sea-water samples were filtered onto 0.2 μm pore size polycarbonate membranes (47 mm, Millipore), which were flash frozen and stored at −80 °C until DNA extraction. 

### 2.2. DNA Extraction, PCR Amplification and High-Throughput Sequencing

Total genomic DNA were extracted from the 0.2 μm polycarbonate filters with the PureLink Genomic DNA Kit (Invitrogen, CA, USA), eluted with 100 μL Tris-EDTA (TE) buffer and stored at −80 °C. Archaeal *amoA* gene fragments were amplified using the barcoded primer Arch-amoAF (5′-adaptor + barcode + STAATGGTCTGGCTTAGACG-3′) and Arch-amoAR (5′-adaptor + barcode + GCGGCCATCCATCTGTATGT-3′) [6]. PCR reactions were performed in a mixture consisting of 1 × PCR buffer, 2 mM MgCl2, 0.2 mM dNTP, 0.4 μM respective primers, 2 units Invitrogen Platinum Taq DNA polymerase (Life Technologies, Carlsbad, CA, USA) and 1μL DNA template. Following the thermal cycle in Francis et al. [6], the triplicates of PCR products were pooled together and purified with the Quick Gel Purification Kit (Invitrogen, CA, USA). For 454-pyrosequencing library preparation, the barcoded purified PCR products were quantified using Quant-iT Picogreen assay (Invitrogen, CA, USA) and mixed at the same concentration following the Rapid Library construction protocols (Roche, 454 Life Science, Branford, CT, USA). Then, samples were loaded into a GS PicoTiterPlate and sequenced using a GS Junior pyrosequencing system according to the manufacturer’s instructions (Roche, 454 Life Science, Branford, CT, USA).

### 2.3. Sequence Processing

The sequence data were analyzed using the microbial ecology community software program Mothur [32]. Quality control was conducted by trimming the low-quality reads (average quality score < 20), those with incorrect length (shorter than 300 bp and longer than 600 bp), those containing an ambiguous base, or containing homopolymers longer than 8 bp. The trimmed sequences were subjected to de-noise process-shhh.seqs with 0.01 sigma value in Mothur. Chimeric sequences were identified through Chimera.uchime and removed using Mothur. The non-*amoA* sequences were removed after the alignment to the *amoA* reference sequences from the National Center for Biotechnology Information (NCBI) database [33]. All remaining high-quality sequences were merged and clustered into operational taxonomic units (OTUs) at 97% DNA similarity as the cutoff value using the make.file and cluster function in Mothur, respectively. Singletons and doubletons were removed from the OTUs table before subsequent analysis. The OTU tables were normalized into the same sequence number per sample. The Good’s coverage indices and rarefaction curves were calculated and generated using Mothur. Similarity among samples was determined based on the Bray-Curtis calculator using the Unweighted Pair Group Method with the arithmetic mean algorithm (UPGMA) in Mothur. Based on a 97% DNA similarity as the cutoff using the normalized OTU table with all observed OTUs, Shannon diversity indices and Margalef richness were calculated using Primer 5 (Primer-E-Ltd, PML, UK). The analysis of similarity (ANOSIM) and Simper analysis were performed using Primer 5 (Primer-E-Ltd, PML, UK). All the raw sequences obtained from this study have been deposited in the NCBI Sequence Read Archive with the accession number PRJNA562607.

The OTUs with ≥ 0.1% mean relative abundance in the sequence dataset were defined as top OTUs, and the rest OTUs were defined as rare groups [34]. To identify the phylogenetic affiliation of the top OTUs, their representative sequences were selected by get.oturep in Mothur based on the distance method and blast (blastN) against the NCBI database [33]. The representative sequences of the top OTUs and the reference sequences from the NCBI database were selected and aligned using MUSCLE version 3.8.31 [35]. A maximum likelihood phylogenetic tree (ML-tree) was generated using Mega 7.0 [36] with 1000 bootstrap replicates based on best model test selection (Tamura three-parameter). The ML-tree was further edited using iTOL [37]. The top OTUs that clustered with the same reference sequences were defined as the same subclades described by Jing et al. and Cheung et al. [15,29].

### 2.4. Analyzing the Relationship between AOA and Environmental Parameters

The relationship between the relative abundance of the AOA subclades and environmental parameters were analyzed with detrended correspondence analysis (DCA) and redundancy analysis (RDA) using Canoco 5.0 [38]. Data from Sta.5_650 m, Sta.2_800 m, Sta.5_800 m, Sta.3_1000 m and Sta.5_3000 m were excluded from the analysis because ammonium and nitrite concentration were not measured. Based on the result of DCA, the length of gradient among 4 axes were lower than 2. Thus, after square root transformation, four environmental variables (ammonium, nitrite, temperature, and dissolved oxygen) were used in RDA to unravel the environmental determinants of WCB subclades (Figure S3: include all AOA subclades in RDA), and depth and salinity were not included in RDA due to high linear dependencies on oxygen and temperature. The exclusion of depth and salinity did not affect the first two axes as they only contributed less than 4.5% of the explained variance. A Monte Carlo permutation test (permutation: 999) was performed to evaluate the significance of the first axis and all axes in the RDA, together with the simple (marginal) and conditional effects of each explanatory variables. OTUs that showed an increase of relative abundance in OMZs were selected and Spearman correlation using IBM^®^ SPSS^®^ Statistics R23.0 between the relative abundance (square root transformed) of these OTUs and DO were calculated.

## 3. Results and Discussion

### 3.1. Hydrographic Conditions and Chemical Profiles in Costa Rica Dome

**Figure 2:**
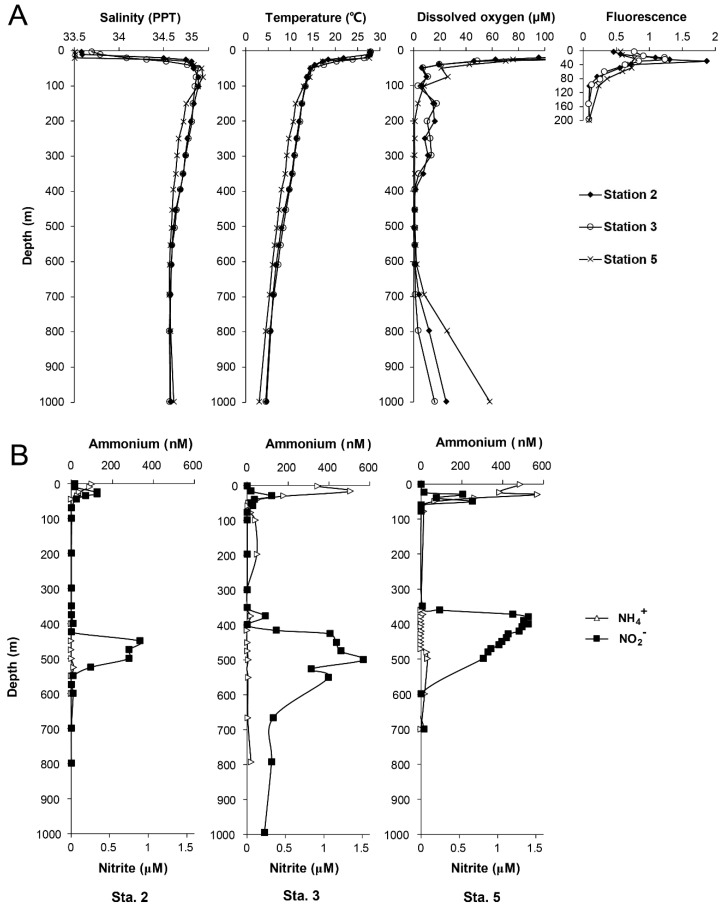
(**A**) Vertical hydrographic profile of salinity, temperature, dissolved oxygen and fluorescence at sampling stations. (**B**) Vertical profiles of ammonium and nitrite concentration at each station.

The vertical hydrographic profiles of all sampling stations were characterized by a sharp increase in salinity and decrease in temperature at ~40 m (Figure 2). At all the stations, DO concentration sharply decreased to 20 μM at 50 m, and further decreased to < 2 μM in core OMZs at a depth of 400–700 m. A distinct primary (PNM) and secondary nitrite maxima (SNM) were observed at all of the stations (Figure 2). The PNM (0.31–0.55 μM) occurred at 20–30 m below the chlorophyll maximum layer (Figure 2A) whereas the SNM (up to 1.5 μM at station 5) occurred within the core OMZ [31]. The ammonium concentration profiles showed maxima ranging between 0.10 and 0.57 μM above or overlapped with the PNM. The largest OMZ was observed in station 3, which was 300 m thick with the largest SNM extending down to 1000 m (Figure 2).

### 3.2. Phylogenetic Diversity of AOA in the CRD

**Figure 3:**
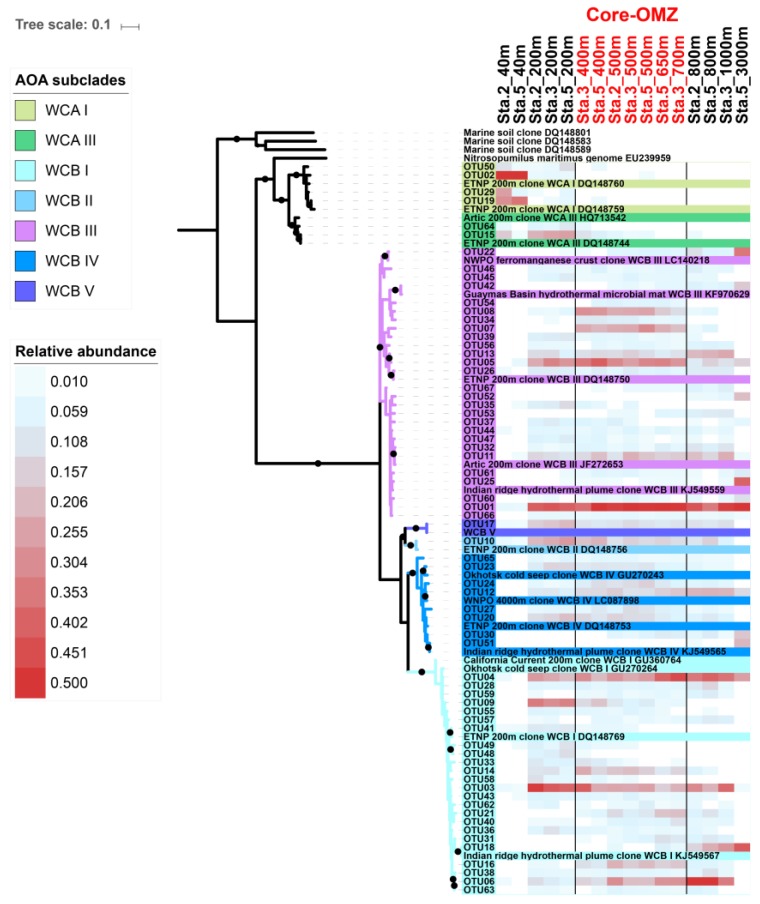
Maximum likelihood phylogenetic tree of archaeal *amoA* gene sequences of abundant operational taxonomic units (OTUs) (mean relative abundance ≥ 0.1%) detected in the Costa Rica Dome and reference sequences. The tree was constructed with best-fit model Tamura three-parameter model (T92 + G + I) and nonparametric bootstrap (1000 replicates), and bootstrap values higher than 60% are displayed as black circles. The ammonia oxidizing archaea (AOA) subclades were defined based on the reference sequences from previous studies [15,29]. The heatmaps were generated with the square root transformed relative abundances of the abundant OTUs.

In total, 16 samples from station 2, 3, and 5 containing 97,543 high quality *amoA* DNA sequences were analyzed in this study, and 797 OTUs were detected. The Good’s coverage indices ranged from 0.971 to 0.999, indicating that the sequencing depth was adequate for reconstructing the AOA communities in the CRD. However, the rarefaction curves did not reach plateau (Figure S1), suggesting that more sequencing effort would be needed to study the rare species. Therefore, only the top OTUs were focused in this study. In total, 67 top OTUs (OTUs with mean relative abundance ≥ 0.1% among samples) were detected. The ML phylogenetic tree showed that the top 67 OTUs were affiliated to the ecotypes, WCA and WCB (Figure 3), which agrees with previous findings that the AOA community in oceanic water mainly consist of WCA and WCB [6,15]. These OTUs were further classified into subclades based on the reference sequences of the studies of Jing et al. 2017 [29] and Cheung et al. 2019 [15]. As a result, WCA consisted of 2 subclades, WCA I [OTU02, OTU19] and WCA III [OTU15, OTU29, OTU50, OTU64], while WCB consisted of 5 subclades, including WCB I [24 OTUs], WCB II [OTU10], WCB III [27 OTUs], WCB IV [8 OTUs], and WCB V [OTU17] (Figure 3). 

Diversity of the AOA community was the lowest in the euphotic layer (40 m) with Shannon indices and Margalef richness ranging from 0.456–0.984 and 1.28–4.11, respectively. The highest diversity was in the upper mesopelagic zone (200 m) with Shannon indices and Margalef richness ranging from 3.1–3.2 and 14.98–19.18, respectively (Table 1) (Figure S2). Previous studies (including in the Arabian Sea, eastern tropical North Pacific and Gulf of California) using quantitative PCR have reported the highest AOA abundance and nitrification activity at similar depths [5,24,25]. In addition to abundance and activity, our results showed the highest AOA diversity in the upper mesopelagic zone, which both WCA and WCB were inhabiting. The tight coupling of organic matter degradation and active nitrification at this depth could substantially draw down the DO concentration [39], which may significantly contribute to the formation of the OMZ. The Shannon indices (2.8–3.2) and Margalef richness (11.83–15.67) were slightly decreased in the OMZ core, while they were still comparable to that at 200 m. This was consistent with a former 16s rRNA gene-based study undertaken during the same cruise that reported the highest diversity of an archaeal community dominated by Marine group I (AOA) in the OMZ core [40], which can be explained by the high tolerance of AOA to oxygen depletion [19,27]. Furthermore, comparing to the shallower OMZ in ETSP (< 300 m depth), where the cooccurrence WCA (64.0%) and WCB (35.0%) contributed substantially to the high diversity of their samples [9], the high diversity AOA in the core OMZ of the CRD mainly consisted of WCB (Figure 3). In addition, the bathypelagic sample from station 5 at 3000 m showed relatively lower AOA Shannon diversity (2.3) and Margalef richness (4.74) than those in the mesopelagic waters (Table 1) (Figure S2). 

### 3.3. Differential Vertical Distribution of AOA Subclades

**Figure 4:**
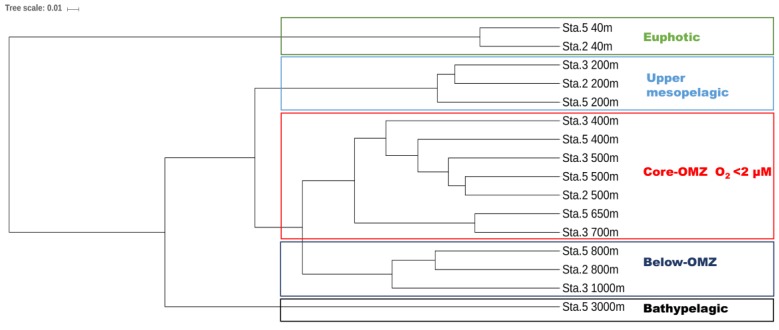
UPGMA dendrogram showing the AOA community relationship based on Bray-Curtis dissimilarity.

**Figure 5:**
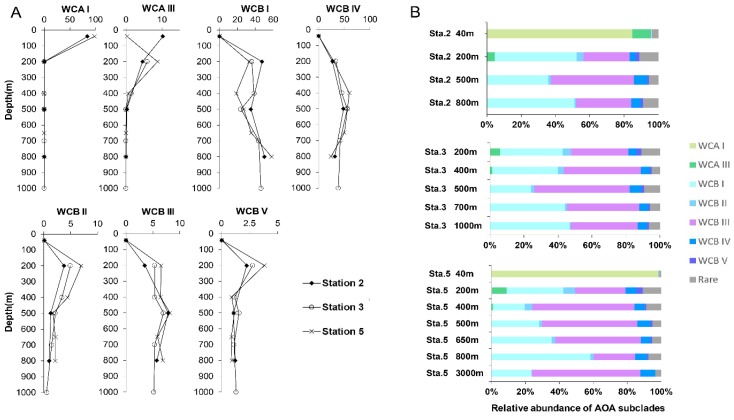
(**A**) Vertical profiles of relative abundance of AOA subclades at sampled stations. (**B**) Vertical profiles of AOA community composition at sampled stations.

The AOA communities were clustered into five distinct groups, corresponding to different layers along the water column: the euphotic layer, upper mesopelagic layer, OMZ core, below-OMZ and bathypelagic layer (Figure 4). As revealed by Simper analysis, the average dissimilarity of the community composition among these groups was 75.5%. In the euphotic zone, the AOA communities was dominated by WCA, in which WCA I and WCAIII contributed 84.8–98.3% and 0.3–11% of the communities, respectively (Figure 5). In the upper mesopelagic layer (200 m), the AOA communities consisted of diverse WCB subclades and a small proportion of WCA III (~5% relative abundance), while the relative abundance of WCA I was negligible. WCB I was the most dominant subclade that accounted for 33.2% to 47.7% of the AOA communities, followed by WCB III, which ranged from 27.1% to 33.8% (Figure 5B). In the OMZ core, the relative abundances of WCA III were further decreased to ~1%, and the AOA communities were almost entirely comprised of WCB subclades. The AOA communities in the OMZ core were dominated by WCB III with a mean relative abundance over 51.2% (Figure 5A), while the second most dominant subclade WCB I accounted for 32.1%. Below the core-OMZ, AOA communities were dominated by WCB I (mean relative abundance = 52.1%) whereas WCB III accounted for an average of 31.9%. Comparing the AOA communities in the OMZ core and the below-OMZ, the dissimilarity of AOA communities was mainly introduced by the distributional difference between WCB I and WCB III, which contributed 17.6% and 19.7% of the dissimilarity, respectively. In the bathypelagic sample from 3000 m at station 5, 64% of the AOA communities belonged to WCB III whereas WCB I accounted for 23.8% of the communities. However, it should be noted that a large proportion of the AOA community at 3000 m deep was comprised of unique OTUs (e.g., OTU 18 of WCB I, OTU 25 of WCB III and OTU51 of WCB IV), which were not abundant at other water layers (Figure 3). 

From another angle, the AOA subclades within the previous defined ecotypes displayed differential vertical distribution patterns. For WCA, WCA I was mainly distributed in the euphotic zone, while the distribution of WCA III extended down to the core-OMZ. This finding supported the previous finding that WCA III can inhabit deeper water than other WCA subclades [15]. For WCB, taking advantage of the steep environmental gradient in the OMZ and the relatively high-resolution vertical profiling of AOA communities along the water column, our results provide the first insight into the differential vertical distributional pattern of WCB subclades. Both WCB I and WCB III were the main AOA in subsurface water, and it was clear that the relative abundance of WCB I was higher in 200 m and below-OMZ than in Core-OMZ where the proportion of WCB III increased. Although the relative abundances of WCB II, WCB IV, and WCB V were much lower than that of WCB I and WCB III, their distribution showed distinct patterns (Figure 5A). The relative abundance of WCB II and WCB V decreased from 200 m all the way down to below-OMZ, whereas that of WCB IV increased from 200 m towards the core-OMZ and below-OMZ.

**Figure 6:**
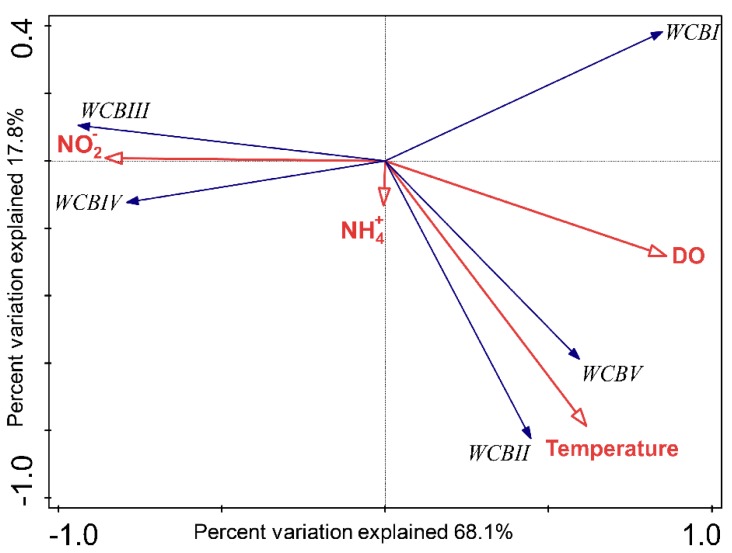
Correlation biplot based on a redundancy analysis (RDA) depicting the relationship between the relative abundance of water column B (WCB) subclades and four environmental factors (ammonium, nitrite, dissolved oxygen concentration and temperature).

As revealed by RDA, the four environmental variables (ammonium, nitrite, DO and temperature) explained 86.4% of the WCB sub-clade variance and the first two axes captured 85.9% of the variance of the dataset (Figure 6). The exclusion of depth and salinity did not affect the first two axes significantly, because they only contributed less than 4.5% of explained variance. DO and nitrite concentration played a critical role on the first axis (Monte Carlo permutation test: F = 8.5, *p* < 0.05), which captured 68.1% of the variance of the WCB community. More importantly, DO contributed significantly with 51.8% (conditional effect: F = 7.5, *p* < 0.05) to WCB community variance, which indicated the importance of the DO level in determining the distribution of WCB subclades along the water column in Costa Rica Dome. Nitrite concentration also contributed 16.1% of the explanatory effect (conditional effects: F = 4.7, *p* < 0.039). In addition, ammonium concentration contributed less than 3.7% of the explanatory effect of the community variance, which may be because of the large variance in ammonium concentration in the OMZ. WCBI, WCB III and WCB IV displayed strong negative and positive correlation to dissolved oxygen and nitrite concentration, respectively, which indicated their preference for a characteristic OMZ environment. WCB II and WCB V showed strong positive correlation to temperature and dissolved oxygen, suggesting that they preferred the conditions in the upper mesopelagic ocean (Figure 6). Hence, our results indicated the differential vertical distribution pattern and environmental determinants of the subclades in the previously defined ecotype WCB [5,6], which were not observed in previous works that quantified the total WCB abundance with same primer set [7,11]. Given that WCB was still uncultivated, in order to further understand the dynamics of WCB subclades in the marine ecosystems, subclade specific qPCR primers are worth designing in future studies. 

### 3.4. Specific WCB Phylotypes Selected by Environmental Conditions in OMZ

Based on the UPGMA dendrogram (Figure 4), the AOA communities in the OMZ core clustered together. Also, the results of the ANOSIM showed that the AOA communities in the OMZ core were significantly different to those in the euphotic zone (*p* < 0.03), upper mesopelagic layer (*p* < 0.01), and below-OMZ (*p* < 0.01). Furthermore, 16 OTUs showed higher relative abundance in the OMZ core (Figure 3), which suggested that the unique conditions in the OMZ core selects these AOA phylotypes. Our results disagree with a previous study in the Arabian Sea OMZ and ETSP OMZ that showed no significant difference between the AOA communities retrieved from oxygenated and anoxic waters [10]. The difference could be caused by the usage of 70 bp sequence to determine AOA phylogeny in the above-mentioned study, which did not provide enough resolution to distinguish different AOA subclades [10]. 

On the other hand, of the 16 OTUs that displayed higher relative abundance in the OMZ, 12 of them have also been detected in the OMZs of other regions (Table 2). For example, OTU07 was absent at 200m but highly dominant in the OMZ core, and its representative sequence was identical to an environmental clone (accession no.: KF512370) from the OMZ in Arabian Sea. OTU08 was mainly distributed in the OMZ core and its representative sequence was identical to an environmental clone (accession no.: EU340498) detected in the OMZ in the Gulf of California (Table 2). Moreover, most of these OTUs were negatively correlated with DO concentration (Table 2). The existence of highly similar AOA phylotypes in the geographically distanced OMZs suggested that some AOA phylotypes may be particularly well adapted to low oxygen conditions and they may play an important role in the biogeochemical cycling in the OMZs. Besides, these results were consistent with previous findings for diazotrophs, in which similar diazotroph phylotypes were also detected in the geographically distanced OMZs [10,15]. Recent findings based on molecular dating accompanied with phylogenomic evidence showed oxygen availability triggered evolution of the terrestrial origin AOA and their expansion from shallow to deep ocean, which indicates oxygen as a critical factor in AOA diversification [41]. More and more studies have reported or proposed mixotrophic life strategies of marine AOA [42,43,44], suggesting that at least some AOA groups do not solely acquire energy via oxidizing ammonia. In order to overcome the oxygen depleted condition in the OMZ, the well-adapted AOA phylotypes might be more heterotrophic because carbon fixation via ammonia oxidation requires extra oxygen molecules. Reji et al. [45] recently introduced ecotype-specific associations between thaumarcheotal and *Nitrospina* phylotypes (nitrite oxidizing bacteria-NOB), providing new insights on microbial association in the role of shaping thaumarchaeal ecotype diversification. Their findings indicated not only the physiochemical determinant and AOA life-strategy versatility, but also microbial association could play important role in AOA diversification and distribution [45]. So far, although several studies have been conducted to examine ammonia oxidation and AOA in marine OMZs [10,31,46], AOA’s adaptive life strategy and role in biogeochemical cycling in OMZs are still unclear. Further exploration should be undertaken using metagenomics that would provide deeper insights than the primer-based approach. 

## Figures and Tables

**Table 1:** Basic information for the *amoA* gene amplicon sequence dataset.

Station	Lon. (W)	Lat. (N)	Depth (m)	DO (μM)	Sequence number	Shannon index	Margalef Richness	Good’s Coverage index
Sta. 2	90.51	8.98	40 200 500 800	19.219 15.87 30.310 11.680	4586 7394 6946 6554	0.984 3.111 3.026 2.838	4.11 19.18 13.39 14.75	0.996 0.981 0.980 0.977
Sta. 3	92.92	9.95	200 400 500 700 1000	10.261 0.651 0.740 1.096 15.920	7438 6569 5413 6569 2435	3.170 2.935 3.162 2.782 2.710	18.51 10.67 15.67 12.08 9.90	0.981 0.984 0.971 0.983 0.981
Sta. 5	87.66	9.35	40 200 400 500 650 800 3000	75.825 7.702 0.640 0.739 1.379 7.850 108.754	5764 6258 7689 7521 7171 5426 3874	0.456 3.239 3.081 2.954 2.839 2.841 2.295	1.28 14.98 15.62 11.83 12.60 11.76 4.74	0.997 0.981 0.982 0.986 0.985 0.984 0.996

**Table 2:** The OTUs that displayed high relative abundance in the oxygen minimum zone (OMZ) core and their highly similar environmental clones from other OMZs. Only the significant Spearman correlation coefficients (*p* value < 0.05) are shown in the table.

OTU No.	Subclades	OMZs	Accession No.	Sequence similarity	Spearman correlation (r_s_) with DO
OTU07	WCB III	Arabian Sea	KF512370	100.00%	−0.782
OTU08	WCB III	Gulf of California	EU340498	100.00%	−0.888
OTU16	WCB I	Gulf of California	KC596407	100.00%	−0.780
OTU06	WCB I	Arabian Sea	KF512353	100.00%	−0.718
OTU14	WCB I	Arabian Se	KF512366	98.73%	−0.815
OTU34	WCB III	Gulf of California	EU340551	100.00%	−0.879
OTU31	WCB I	Gulf of California	KC596421	100.00%	
OTU33	WCB I	Gulf of California	KC596422	99.68%	
OTU54	WCB III	Gulf of California	EU340552	100.00%	−0.763
OTU30	WCB IV	Gulf of California	KC596405	100.00%	
OTU11	WCB III	Gulf of California	KC596420	100.00%	
OTU57	WCB I	Arabian Sea	KF512363	100.00%	−0.619
OTU21	WCB I	CRD	This study		−0.734
OTU24	WCB IV	CRD	This study		−0.825
OTU27	WCB IV	CRD	This study		−0.679
OTU61	WCB III	CRD	This study		−0.837

## Supplementary Materials

The following are available online at https://www.mdpi.com/2076-2607/7/10/453/s1, Figure S1: Rarefaction curve of sequencing sample, Figure S2: Vertical profile of A. Shannon diversity indices and B. Margalef richness of Costa Rica Dome (CRD). Figure S3: Correlation biplot based on a redundancy analysis (RDA) depicting the relationship between the relative abundance of AOA subclades and 4 environmental factors (ammonium, nitrite, dissolved oxygen concentration and temperature).
