# Supplementary figures and images for: Differential Distribution and Determinants of Ammonia Oxidizing Archaea Sublineages in the Oxygen Minimum Zone off Costa Rica
Source: Microorganisms. 2019 Oct 15;7(10):453. doi: 10.3390/microorganisms7100453 (PMC6843251; doi:10.3390/microorganisms7100453)

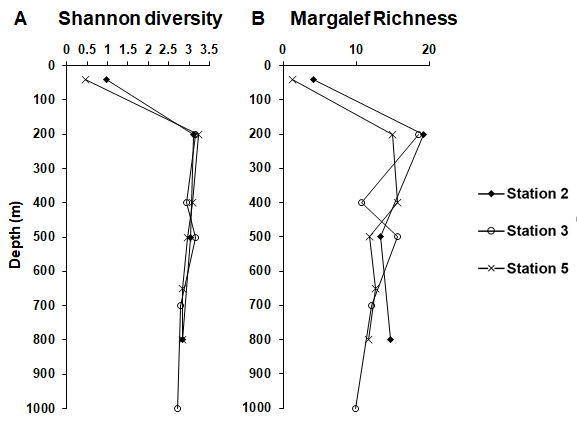

Supplement: Supplementary file 1 [file microorganisms-07-00453-s001.zip › Figure S2.tif]

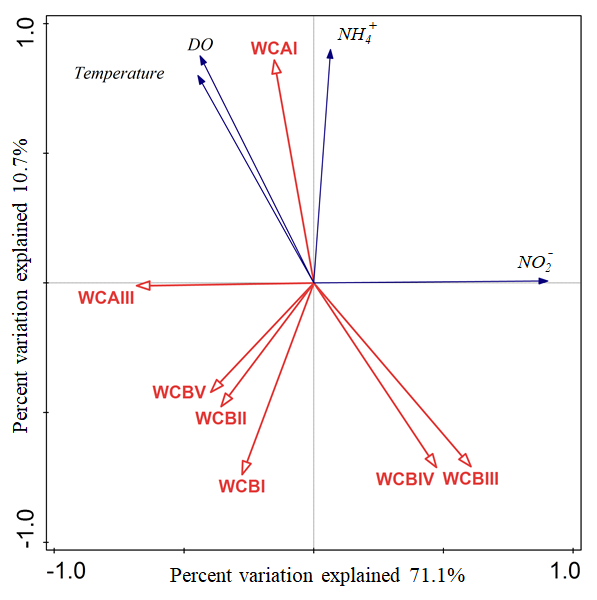

Supplement: Supplementary file 1 [file microorganisms-07-00453-s001.zip › Figure S3.tif]

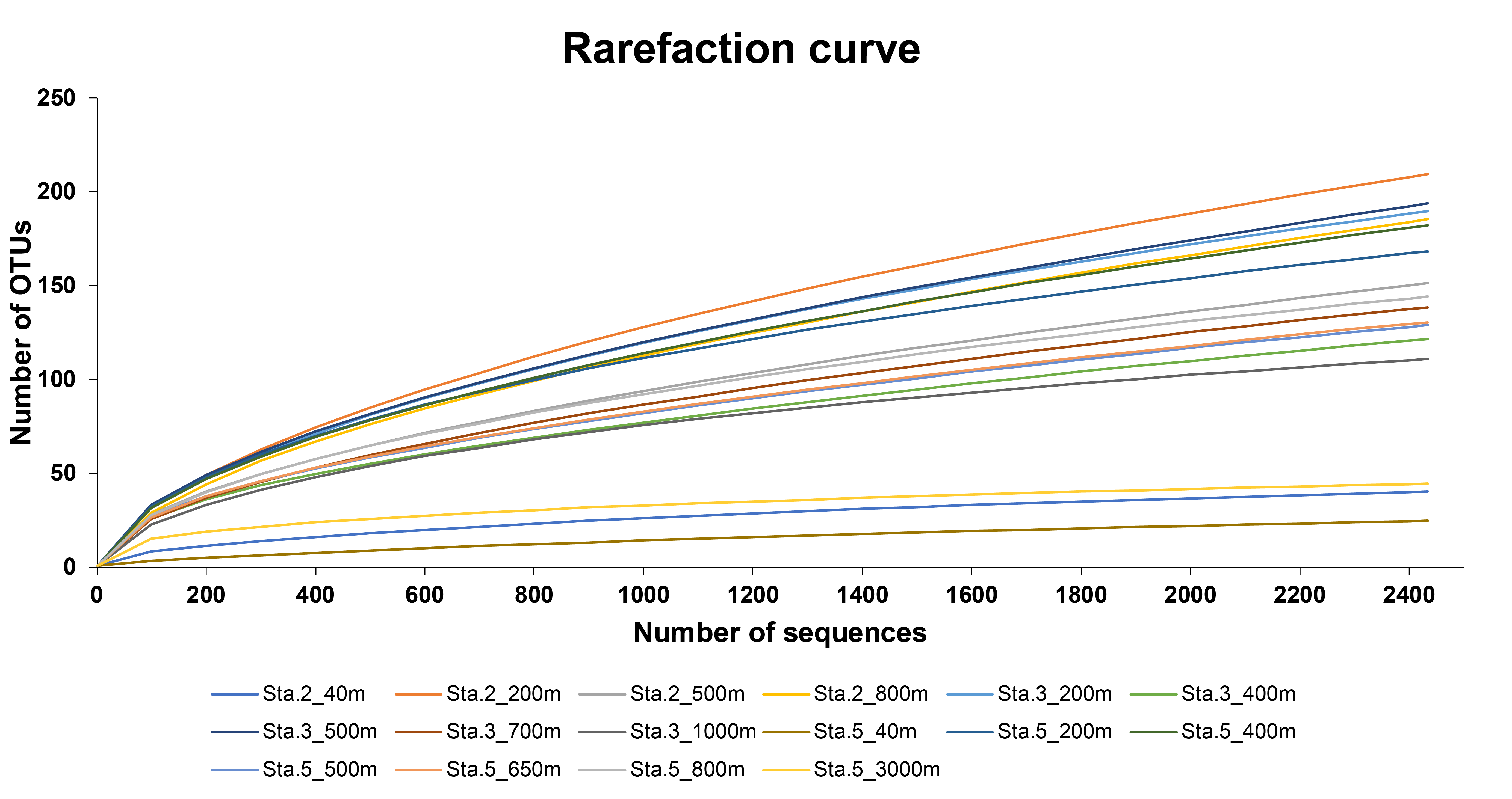

Supplement: Supplementary file 1 [file microorganisms-07-00453-s001.zip › Figure S1.tif]
